# Conceptualising morally permissible risk imposition without quantified individual risks

**DOI:** 10.1007/s11229-022-03888-4

**Published:** 2022-10-03

**Authors:** Susanne Burri

**Affiliations:** Department of Philosophy, Faculty of Humanities, University of Konstanz, Constance, Germany

**Keywords:** Risk, Permissible risk imposition, Probability, Frequentism, Subjective interpretations of probability, Reference class problem

## Abstract

We frequently engage in activities that impose a risk of serious harm on innocent others in order to realise trivial benefits for ourselves or third parties. Many moral theories tie the evidence-relative permissibility of engaging in such activities to the *size of the risk that an individual agent imposes*. I argue that we should move away from such a reliance on *quantified individual risks* when conceptualising morally permissible risk imposition. Under most circumstances of interest, a conscientious reasoner will identify a gap between the factors they deem potentially relevant to the riskiness of an agent’s behaviour, and the factors they are reasonably able to quantify. This then leads a conscientious reasoner to conclude that they cannot, in good faith, come up with a quantitative risk estimate that is genuinely tailored to the agent’s particular situation. Based on this, I argue that principles of morally permissible risk imposition fail to provide us with practical guidance if they ask us to take into account our agent-specific risks in a quantified manner. I also argue that principles of permissible risk imposition which appeal to quantified individual risks implausibly imply that it is frequently indeterminate whether engaging in some risky activity is morally permissible. For both of these reasons, I contend that principles of morally permissible risk imposition should make no reference to quantified individual risks. They should instead acknowledge that any quantitative estimates that an agent might usefully be able to consider will likely not be tailored to the agent’s idiosyncratic situation.

## Introduction

We know that many of the activities that we engage in while going about our daily lives might threaten significant harm to innocent others. Examples of such *risk-imposing activities* include driving a car, hosting a barbecue, or walking a dog of a “dangerous” breed. On most occasions where we decide to pursue such activities, we aim to reap only trivial benefits for ourselves or a few others, and we could, in this sense, easily refrain from engaging in them. At the same time, the harm that we might threaten is potentially serious. This raises the question of what makes such risk-imposing behaviour at least sometimes morally permissible. Importantly, this is a question located at the level of *subjective* morality. As I employ the term in this paper, an action is *subjectively morally permissible* just in case an agent is not morally required to refrain from it in light of the evidence available to her at the time of acting.^1^ The counterpart to subjective permissibility is objective permissibility. Objective permissibility captures what an agent would not be required to refrain from doing if, at the time of acting, she had access to all the morally relevant facts.^2^ Risky activities are not, in general, a topic of interest from the point of view of objective morality, as risky activities are rendered risky largely by our uncertainty, at the time of acting, whether we will cause harm if we choose to engage in them.

Many existing accounts of the moral permissibility of engaging in a risk-imposing activity appeal to how sizeable a risk an agent imposes to explain why engaging in a risk-imposing activity need not be morally objectionable even if the agent seeks to realise only trivial benefits. Their guiding thought is that if the probability of harm occurring is tiny, then the size of the risk—i.e., the probability that harm will occur multiplied by the disvalue of the harm—that an agent imposes on her potential victims is small, too, even if the injuries that her victims might suffer are potentially serious. The thought continues that if trivial benefits are highly likely to occur, then their moral significance can outweigh the significance of a sufficiently small risk of harm.

In this paper, I argue that we should move away from a reliance on such *quantified individual risks* when conceptualising the moral permissibility of engaging in risk-imposing activities. On the face of it, appealing to such risks introduces a clear reference point in an area of moral philosophy that is otherwise fraught with complexity. But I argue that this impression is mistaken. Admittedly, as agents who are interested in performing some activity, we will usually have access to trustworthy data that enables us to come up with quantitative estimates that are related to the activity we are interested in pursuing. We might, for example, be able to estimate in good faith what risks an average member of our society imposes on others by engaging in the relevant activity. At the same time, we will usually be confident that our own situation is characterised by an idiosyncratic combination of risk-relevant factors that we lack the tools to quantify. We will thus frequently have access to some relevant quantifications, while at the same time appreciating that these are not tailored to the risk profile characterising our particular circumstances.

The fact that there are important practical limits to what we are reasonably able to quantify renders quantified individual risks an ill-suited feature of subjective moral principles—or so I argue in this paper. As I see it, subjective moral principles serve two main purposes. Some aim primarily at providing helpful guidance to well-intentioned agents. The point of such principles is to build a bridge between objective moral principles and an agent’s deliberative situation, where this situation makes it unworkable for the agent directly to apply objective principles.^3^ Other subjective principles are not predominantly concerned with guiding action. Their main aim is to identify the features of an action that render the action subjectively right or wrong, quite independently of whether these features are directly actionable. Whatever its primary function, I claim that a subjective principle for the permissibility of engaging in risk-imposing activities should not make any reference to quantified individual risks. If it is the principle’s main purpose to guide action, the difficulties we encounter when trying to quantify agent-specific risks render an appeal to such risks practically unhelpful, thus invalidating the principle. Where a principle’s main role is explanatory, its reliance on quantified individual risks implausibly implies that it is frequently indeterminate whether engaging in a risk-imposing activity is morally permissible. Subjective moral principles do better, I propose, if they proceed from the insight that quantifications of riskiness will usually not be agent-specific, but will be tied to a reference class that is broader than the one we deem appropriate for any particular agent. This does not mean that the principles—and our thinking about what constitutes morally permissible risk imposition—should aspire to shun quantifications. It merely means that we have to be careful in our interpretation of the quantitative elements that we continue to rely on, and that their limitations can sometimes make it appropriate to complement them with non-quantitative insights.

The remainder of this paper is structured as follows. In Sect. 2, I discuss how existing accounts of morally permissible risk imposition rely on quantified individual risks. The theories that I focus on are maximising act utilitarian and deontological in nature, but my analysis doubtlessly generalises to other approaches. I focus on the approaches just mentioned because they usefully illustrate the two main purposes that subjective moral principles serve. In Sect. 3, I argue that accounts that appeal to quantified individual risks fail to provide practical guidance to well-intentioned agents (Sect. 3.2), and home in on a feature of actions that is not plausibly of moral significance (Sect. 3.3). In Sect. 4, I sketch how—in a central set of cases—we might conceptualise morally permissible risk imposition without appealing to quantified individual risks. Section 5 concludes.

## Examples of accounts that rely on quantified individual risks

### Maximising act utilitarian theories

Maximising act utilitarian theories are consequentialist moral theories that evaluate the goodness or badness of different states of affairs in terms of the total welfare the states contain. According to maximising act utilitarian theories, it is objectively morally permissible to perform an action just in case doing so leads to a state of affairs that contains a sum total of welfare that is at least as large as the sum total of welfare connected to any other state of affairs the agent is in a position to bring about. As agents, we are usually unable to work out what we would have to do for our actions to be morally optimific in this way. But this need not speak against maximising act utilitarianism. If there are strategies we can follow that help us promote the best possible consequences reasonably reliably, this suffices for maximising act utilitarianism to provide us with—albeit indirect—practical guidance.^4^ To put this point more generally, a moral principle is not fatally flawed merely because the right- or wrong-making features that it identifies fail to be directly action-guiding. Only if we are unable to supply action-guiding strategies that help well-intentioned agents abide by the principle may we appropriately start to worry about the principle’s adequacy.

Maximising act utilitarians standardly defend a subjective principle tied to the notion of *expected value* to help agents act in conformity with the objective demands of their theory.^5^ More precisely, they tend to argue that it is subjectively permissible to choose an alternative for action just in case its *expected value* is at least as high as the expected value of any other perceived alternative. The expected value of an action is the sum of the estimated values of the different outcomes the action might bring about, with each outcome discounted by its probability of occurring if the action is performed.

Applied to the problem of risk-imposing activities, this principle clearly makes reference to quantified individual risks. To calculate the expected value of an activity such as dog-walking, it is necessary to put a number on the risk that one thereby imposes, i.e., to estimate quantitatively the probabilities with which harmful outcomes might occur depending on what one does, and to multiply these probabilities with the estimated quantified disvalues of the harmful outcomes.

### Deontological moral theories

Maximising act utilitarians put forward subjective principles to provide well-intentioned agents with the tools they need to track the demands of objective morality. This entirely instrumental way of thinking about subjective principles is widespread among consequentialists, and it has supporters among deontologists as well.^6^ But there are dissenting voices also. Some moral theorists believe that morally relevant considerations are located not only at an objective level, but also—and, in a sense, even primarily—at a subjective level.^7^ As I understand it, the guiding thought behind this rather different way of looking at things is that the rightness or wrongness of an action is intimately bound up with the evidence that is available to an agent at the time of acting. In cases where an agent has access to all the morally relevant facts, we find ourselves in the realm of objective morality, but this is really just a special case of subjective morality, namely one where no pertinent information is out of reach. Where an agent does not have access to all the morally relevant facts, what it is right for her to do is specified in terms of the available information, and need not be informed by what it would be right for her to do if she had access to all the relevant facts.^8^

Where subjective moral principles are understood in this non-instrumental way, they are put forward as independent moral precepts that aim to identify an action’s right- or wrong-making features in light of the evidence available to an agent at the time of acting. Such principles are not fatally flawed if they fail to guide action, as this is not their primary point. As long as there are heuristics available that help agents abide by the principles, the principles are able—albeit indirectly—to guide behaviour. What instead makes or breaks such principles is their ability to identify morally relevant considerations, thus helping us explain and justify claims about right and wrong actions. Crisply put, the principles’ main point is not to help us get things right, but to clarify what it would mean for us to get things right.

When deontologists consider what might make engaging in risky activities morally permissible, they frequently appeal to quantified individual risks.^9^ Of course, deontological moral theorising is extremely diverse, and the subjective principles put forward differ in important ways. What matters for my purposes, however, is the fact that the principles frequently make reference to the probability with which certain outcomes will occur if a particular action is performed.

As a first example, consider Robert Nozick, who ponders “how slight a probability of harm that violates someone’s rights” an agent needs to impose to violate rights through their risk imposition.^10^ With this way of looking at things, Nozick takes it as a given that engaging in a risk-imposing activity violates rights and is for this reason morally impermissible once the probability of causing harm passes some numerical threshold.

More recently, numerous contractualists have considered the problem of morally permissible risk imposition, and many have defended so-called “hybrid” or “*ex ante*” frameworks according to which it matters how likely it is that individuals will suffer harm if some action is performed.^11^ In a telling passage, James Lenman argues that agents who are engaged in risk-imposing activities act permissibly only if they take *sufficient precautions*, which they do only if the risks they impose on others are sufficiently small. As Lenman puts it:

Here is McTweedle enjoying a country drive, thereby imposing a one in a million risk of death on the locals. And here am I, gaining a comparable amount of private pleasure by practising my favourite Sunday pursuit of setting off small, controlled explosions ...in my kitchen, thereby imposing a one in ten risk of death on my neighbours. Suppose we are both being really, really careful and that these probabilities would each be much higher were we less so. Well, that is certainly to be commended, but the fact very plausibly remains that what I am doing is impermissible and what McTweedle is doing is not and that this ...is due at least in large part to the fact that the probability of harm in his case ...is so very low and the probability of harm in my case ...so very high.^12^

This passage is telling because Lenman simply *stipulates* what risks he and McTweedle impose on others through their respective pursuits. This practice is widespread in moral theorising, and it suggests that quantified individual risks are regarded as both unproblematic and informative, and thus as the sort of consideration that we might usefully appeal to when discussing what might make it permissible to engage in risk-imposing activities.

As a final example, Seth Lazar argues that a principle he coins COST captures the “necessary and sufficient conditions for an act’s being permissible in light of an agent’s uncertainty ...”^13^ Among other things, COST appeals to the “expected choiceworthiness” of an action, i.e. the “probability-weighted average of the objective rankings of the possible outcomes.”^14^

## Problematising quantified individual risks

Suppose you endorse an account of morally permissible risk imposition that appeals to quantified individual risks. For simplicity, let us assume that the account that you endorse makes reference to the expected harm of engaging in a risk-imposing activity. You want to make sure not to break any moral rules. Before you take your Pit Bull Faustus on his next walk, you therefore try to estimate what risks, i.e., how sizeable an expected harm, walking Faustus imposes on those around you. How can you arrive at such an estimate? On the one hand, you have to appropriately quantify the badness of the harmful outcomes that your dog walking might bring about. On the other hand, you have to judge the probability with which your dog walking might, in fact, result in these outcomes. While both of these tasks come with formidable difficulties, my focus in this paper is entirely on the second.

In Sects. 3.2 and 3.3, I will discuss the *epistemic* and *metaphysical* issues we face if we take seriously the idea that the permissibility of engaging in a risk-imposing activity depends at least in part on how likely it is that engaging in the activity will cause harm. The epistemic issues are worrisome primarily if a subjective rule aims to provide guidance to well-intentioned agents. The metaphysical issues are worrisome first and foremost if a subjective rule is put forward as an independent moral precept that aims to capture the morally relevant features of an action in light of the evidence available to an agent at the time of acting.

Before discussing these difficulties, however, we first have to narrow down what we mean by the *probability* with which some suitably described outcome will be brought about if an action is performed. In the absence of such a clarification, we cannot informatively discuss how you might conscientiously go about assigning probabilities to the harmful outcomes that you might bring about by walking Faustus.

### An interpretation of probability suitable for subjective moral principles

Interpretations of probability are commonly divided into objective and subjective ones. Objective interpretations tie probability to mind-independent features of the world; subjective ones conceptualise it as mind-dependent degree of belief, the technical term for which is *credence*. Of the main interpretations of probability, all face important challenges, and their suitability depends on the context.^15^

Where subjective moral principles make reference to probability, I suggest that we most suitably settle for an *objectively constrained subjective interpretation* of the term. Objective interpretations are unsuitable because they fail to limit themselves to whatever evidence happens to be available to a particular reasoner. This makes them ill-aligned with subjective moral principles, of which it is true by definition that they respect the epistemic constraints an agent faces. To see this more clearly, consider frequentism, the objective interpretation of probability most promising for our purposes. According to frequentism, the probability of an event occurring is the *relative frequency with which the type of event occurs in some relevant reference class*.^16^ Suppose that, over the past ten years, people in your country have walked their dogs for an average of 365 million dog walking hours per year. Further suppose that, for the same period, the yearly average of dog-walking related accidents involving serious harm was 365. If we regard what happened within your country over the past 10 years as the relevant reference class for the risk that you impose by walking Faustus, it follows that, according to frequentism, there is a one in a million probability that you will cause serious harm by taking Faustus for an hour-long walk. Crucially, this holds true even if you lack access to national dog-walking statistics, for example because the relevant data were never collected.

Subjective interpretations of probability make no reference to information that may be out of an agent’s reach, and this makes them more appropriate for our purposes. Having said that, objectively unconstrained subjective principles make only insufficient appeal to the evidence that an agent does have access to. Consider subjective Bayesianism, which claims that the probability of an event occurring is simply the coherent credence^17^ that an agent assigns to the truth of the proposition that the event will occur. Subjective Bayesianism is not, in general, able to rule out that the probability of an event occurring varies widely for different reasoners who have access to the same evidence, as the reasoners’ judgements need not be responsive to the evidence in ways that we would normally deem appropriate.^18^ In Alan Hájek’s words, “[t]he epistemology [implied by subjective Bayesianism] is so *spectacularly* permissive that it sanctions opinions that we would normally call ridiculous.”^19^ This renders subjective Bayesianism an unsuitable interpretation of probability for subjective moral principles, irrespective of whether they aim to guide action or attempt to capture the morally relevant features of an action. If the former, the point of the principles is to help agents track an objective reality that is at least partly hidden from view. Plausibly, this is possible only if the principles require that agents make good use of whatever information they have access to. If the latter, it strains credulity that a judgement largely unconstrained by an agent’s evidence should be a morally relevant feature of the agent’s action as assessed from an evidence-relative perspective.^20^

Based on the identified problems, I propose that we narrow the set of plausible interpretations of probability for our purposes to *objectively constrained subjective interpretations*. Such interpretations stipulate that probabilities are essentially coherent credences, but add to this the thought that they are the credences of a reasoner who adequately takes into account the available information. According to the specific interpretation that I propose now and will employ in the remainder of this paper, *the probability of an event occurring in light of some body of evidence is the coherent credence that a conscientious and statistically well-versed reasoner would assign to it in light of this body of evidence*. On my understanding of the term, a conscientious and statistically well-versed reasoner (i) spends a significant amount of time looking for, and processing, potentially relevant data and studies; (ii) competently sifts through available information, making use of evidence that is able to inform their credences, setting aside what is irrelevant or of questionable quality; (iii) appropriately interprets information as it pertains to the particular situation they are interested in.^21^ When we try to come up with probability estimates as decidedly imperfect reasoners, it should be our aim to track the judgements of such an expert. What I will argue in the next section is that once we try to emulate this idealised figure of the conscientious and statistically well-versed reasoner, we realise that there is no satisfactory strategy for doing so.

### Epistemic issues

Let us return to the Pit Bull example. As a dog owner who wants to know what risks you impose on others by walking Faustus, how might you aim to track the credences of a statistically well-versed expert? It seems clear that, to begin with, you would want to obtain information that helps you quantify both (i) how much dog walking there is in your society; (ii) how much of this dog walking tends to result in an accident. A solid estimate of these two figures allows you to calculate the relative frequency with which dog walking results in an accident in your society, and it seems reasonable to suppose that *some* such relative frequency is what a conscientious and statistically well-versed reasoner would aim to establish.^22^ While frequentism is thus ill-suited as a direct interpretation of probability for our purposes, its insights nevertheless play a key role in determining how we should make use of the available evidence. Suppose, then, that you look for relevant data, and are able to locate national accident statistics for the past 10 years. The statistics show a breakdown of accidents by category, including a category that is labelled “dog-related.” As far as you can tell, this includes *all* reported dog-related accidents, including those that occur in the home, in gardens, or at the vet. In a separate search, you also find an estimate of the number of dogs owned in your country, but are unable to locate information about the amount of time they spend on walks. You do know, however, that you walk Faustus for roughly two hours each day. If you multiply these two hours by the 365 days that are in a year, and multiply this number by the estimated dog population, you arrive at an estimate for the national annual dog-walking hours. If you divide the number of annual dog-related accidents by this estimate of dog-walking hours, you arrive at a first estimate of accidents per hour.

How should you further refine this ballpark figure, if at all? What would a conscientious and statistically well-versed reasoner do? You know that your current figure overestimates walking-related accidents in one sense, as the accident statistics are not limited to walks. You also have a hunch that you might underestimate walking-related accidents because you think that the average dog owner might walk their dog for much less than two hours per day. In addition, you think that *your* dog walking is affected by factors that raise its riskiness compared to other people’s dog walking (Faustus is of what is often referred to as a “dangerous” breed; you live in a densely populated area), but also by factors that lower your idiosyncratic risk (you nearly always keep Faustus on a leash even though there is no general leashing requirement where you live; Faustus is not aggressive; you are an experienced dog owner). Finally, you have not done anything to assess the quality of the national statistics that you draw on. Would a conscientious reasoner look for more information? Would they do more to check the quality of the data they draw on?

As someone trying to emulate the judgement of an expert, three broad strategies seem available to you. First, you might decide to **look for additional evidence**. Maybe you can find dog accident statistics for the densely populated city that you live in, not merely national statistics. Maybe there are statistics specifically for “dangerous” dog breeds. Maybe there are studies that allow you to quantify the relevant causal effects of being an experienced dog owner, or of keeping your dog on a leash.^23^ The general idea of this first strategy is to keep looking for evidence until you feel you have done what you can to quantify all of the factors that, on careful reflection, strike you as pertinent. The main problem with this strategy from an action-guiding point of view is that it burdens you with a time-consuming and cognitively demanding investigative task. Not only do you have to look for—and carefully evaluate—data and studies with respect to all the risk-imposing activities that you might be interested in pursuing, but you also have to acquire a solid understanding of statistics to assess the quality and trustworthiness of the information you are able to locate. Admittedly, you will not have to engage in such a research task each time you consider engaging in a risky activity. Once you have done a thorough assessment of an activity, you should be able to come up with context-specific estimates much more quickly. Still, the burdensome task of the initial assessment remains, and you would, on this first strategy, have a duty to be on the lookout for new data and new studies that pertain to the risky activities that you engage in. You would also have to conduct additional research whenever you had reason to assume that your risk characteristics had changed (in the Pit Bull example, this would apply if e.g., you acquired a second dog and started to walk your dogs jointly). This undermines the idea that we provide agents with useful practical guidance if we tell them to consider what quantified individual risks they would impose by pursuing some alternative.

Based on the weaknesses of this first strategy, you might decide that you should have to incur only more limited costs when coming up with a probability estimate. You might thus decide to **wing it**. The general idea of this second strategy is not to aim at gathering evidence until you have conscientiously quantified the effects of all the factors that you deem potentially relevant, but to stop gathering evidence once you have found *some* relevant data, taking into account the special characteristics of your case in a rough-and-ready manner. If you divide national data on dog-related accidents by your estimated number of national annual dog-walking hours, this yields a 0.00000137 probability that an accident will occur during one hour of dog walking. On the “winging it” strategy, you multiply this probability by ten because Faustus is a Pit Bull, roughly because “dangerous breed” seems a significant factor, and because multiplying something by ten seems to increase it significantly. Based on similar reasoning, you multiply the probability by two to account for the fact that you live in a densely populated area, and then you divide it by five, and then by two, and again by two, because you are an experienced dog owner who owns a non-aggressive dog who is kept on a leash. Voilà.

Compared to the first strategy, the obvious advantage of this second strategy is that you will reach a verdict within a useful time frame. The obvious disadvantage is that you have no clue, really, whether your quantifications are similar to the ones that a conscientious reasoner would arrive at (if they would, indeed, arrive at a quantification at all; see Sect. 3.3). There might, for example, exist solid evidence that owning a dog of a dangerous breed increases the risk of a report-worthy accident not tenfold, but a thousandfold. In this way, you have little reason to assume that the guesstimates that you come up with track relevant data that might in principle be available. Relatedly, you have no reason to assume that your risk assessment will be shared by others, and no evidence to cite to convince others that you have made the “right” adjustments to the population-level data to take into account your idiosyncratic characteristics. There is also a danger that you will engage in motivated reasoning. Specifically, if you are keen on a particular pursuit, you will likely assess the way in which you would engage in it as really quite safe. My own anecdotal evidence suggests, for example, that many dog owners are convinced that *their* dog would *never* attack anyone.^24^

Trying to avoid the problems of the first two strategies, you might decide simply to **stick with a rough evidence-based estimate**. If you adopt this third strategy, you make neither a conscientious nor a very casual attempt to quantify the effects of all the factors you deem potentially relevant. In the Pit Bull example, once you find the national statistics that lead you to conclude that there is a 0.00000137 probability of an accident per hour of dog-walking, you simply stick with this number, making no further adjustments. Compared to the second strategy, this strategy leaves less room for biased reasoning. But, as with the second strategy, you have little reason to assume that your guesstimate is close to what a conscientious and statistically well-versed individual would come up with. You also know that you do not even make an attempt to quantify factors that seem relevant to your agent-specific risk. In this sense, you knowingly replace the notion of a *quantified individual risk* employed by the subjective moral principle that you are trying to follow with something like the *average societal risk of the activity in question*, assuming that such a replacement is admissible. But the two notions are very different, and you will usually have no reason to assume that they happen to coincide in your particular case.^25^ Also, if we are dealing with a moral principle whose main point it is to guide action, it seems that no such replacement should be necessary—whatever considerations the principle appeals to should be quite readily accessible to you.

Based on this, I conclude that if we understand probabilities as credences that a conscientious and statistically well-versed reasoner would adopt in light of the available evidence, then subjective moral principles that appeal to quantified individual risks fail to be action-guiding. If an agent makes a good faith attempt at quantifying in an evidence-based manner the effects of all the factors that she deems potentially relevant, she faces a cognitively demanding and possibly quite open-ended task. If the agent makes no such serious attempt, she has insufficient reason to regard her guesstimates as approaching the credences of an expert, which the subjective principle implies are relevant.

On reflection, there is something unsurprising about this conclusion. When we consider engaging in a risk-imposing activity, it would be highly unusual for most of us to try to attach numerical probability estimates to the occurrence of possible harmful outcomes. Even for the risk-imposing activities that we regularly engage in, I doubt that many of us have considered, in detail, how likely it is that we will cause harm through them. The more quantitatively informed among us might have some understanding of the society-wide harms associated with some activity, but are unlikely to have made a serious attempt at quantifying their idiosyncratic deviation from the societal average. In short, it is decidedly not the case that quantifying individual risks has emerged as something like a best practice.^26^ This makes it not entirely surprising, then, that appealing to quantified individual risks does not provide well-intentioned agents with useful guidance.

### Metaphysical issues

The exclusively epistemic issues discussed so far need not be an issue for subjective moral principles that are put forward as independent moral precepts. As long as we can supplement such principles with action-guiding heuristics that enable well-intentioned agents to abide by the principles, the principles are not invalidated by the fact that an imperfect but well-intentioned agent cannot follow them directly. It is, however, a serious issue for such principles if we have reason to doubt the adequacy of the right- or wrong-making features they identify. In this section, I argue that this is the case for subjective moral principles that appeal to quantified individual risks. According to the argument that I put forward, quantified individual risks are an implausible right- or wrong-making feature from a subjective perspective because they are frequently indeterminate.

Consider again the Pit Bull example, but try to imagine now that you are not an imperfect agent attempting to emulate a conscientious and statistically well-versed reasoner, but that you qualify, instead, as such an idealised reasoner. On careful reflection about the nature and causes of accidents related to dog-walking, you conclude that the riskiness of your dog-walking is at least potentially affected by (i) Faustus’ breed (he’s a Pit Bull, which is considered a “dangerous” breed); (ii) Faustus’ character (solidly-good natured); (iii) your level of experience with dogs (advanced); (iv) the extent to which you keep Faustus on a leash (95%); (v) the population density of the areas where you walk Faustus (high). Alas, search as you will, you find reasonably reliable data only about dog-related accidents in general, plus some studies that allow you to make a rough guess about the significance of Faustus’ breed. You thus find yourself in a situation where, *on the available evidence, which includes your general understanding of how dog-related accidents are brought about, you deem more factors plausibly relevant than the evidence allows you to quantify*. I project that this is an extremely common predicament for a conscientious and statistically well-versed reasoner to find herself in; in fact, I project that departures from this predicament most likely qualify as exceptions to a general rule. These projections are based on the fact that gathering, maintaining, and making available high-quality data is both very costly and very demanding. In addition, the task of estimating what causal impact a specific factor has on some phenomenon is fraught with difficulty. In light of this, it is not surprising that for many factors where, based on our general understanding of the issue, we have a plausible hypothesis about how the factor might be related to some variable of interest, no one has made a serious effort to test the hypothesis and to quantify whatever effect might be found. All things considered, this lack of quantification may well be morally desirable. After all, our resources are scarce, and it is not obvious that it would be morally optimal to allocate a significantly increased amount of resources to data collection and the testing of causal hypotheses.

In situations where an idealised reasoner is unable conscientiously to quantify all the factors they deem relevant to some probability, I contend that they would generally refuse to assign a number—or a suitably narrow range of numbers^27^—to this probability. Based on the recognition that they are unable to take into account everything they deem potentially relevant, they would regard it as unduly speculative to settle for an estimate. Of course, if we force the reasoner to give us their “best guess”, or if we simply assume that they must have arrived at such a best guess and go on to infer it from their behaviour, we may be able to extract a number, or some sufficiently narrow interval, from our idealised reasoner. But this quantification would not, then, represent the reasoner’s considered judgement. As Hugh Mellor puts it, “[measuring credences] by forcing a man to choose betting odds, for example, *presupposes* that he has [credences] which the chosen odds measure;”^28^ it does nothing to *establish* that the man does, in fact, hold any such beliefs. In a nutshell, then, I am claiming that the probability of harm occurring is frequently indeterminate because an idealised reasoner would frequently prefer to withhold judgement.

Of course, it need not always be true that an idealised reasoner would decide to withhold judgement. Sometimes there might be no gap between the factors the reasoner deems potentially relevant and the ones whose effects they are able to quantify based on existing data and studies. At other times, such a gap may exist, but the reasoner may nevertheless be able to come up with a useful estimate. In the moral context that is of interest to us, it may, for example, frequently suffice for the moral permissibility of an action that the risk imposed through it does not pass a certain threshold.^29^ And whether this is the case can sometimes be established even if not all potentially relevant risk factors can be quantified. To see this, consider a situation where a conscientious and statistically well-versed reasoner has access to data about a population with mixed risk characteristics. To go back to our dog walking example, suppose the reasoner has access to national dog-walking statistics which show that, on average, there was one serious accident per one million dog walking hours. Further suppose that it is morally permissible to walk one’s dog just in case one imposes a risk that is no higher than this national average. Lisa is a very experienced dog handler who lives in the countryside. She owns a friendly Labrador Retriever whom she always keeps on a leash. With respect to the risk that Lisa imposes through her dog walking, a conscientious reasoner may justifiably conclude that the national average constitutes an upper bound for it, as *all* of Lisa’s relevant characteristics make her a particularly low-risk dog walker. Lisa may thus justifiably conclude that she imposes no undue risks by walking her beloved pet. As soon as we have reason to assume, however, that an agent’s idiosyncratic risk factors pull in opposing directions—making her actions safer than some relevant population average in some respects, but less safe in other respects—the relevant population average can no longer serve as a trustworthy upper (or, for that matter, lower) bound on the agent’s idiosyncratic risk.^30^

If what I have argued is correct, then the risk of harm that a particular agent imposes on others by engaging in some activity is frequently indeterminate, and it will thus frequently be indeterminate whether some quantified individual risk is “sufficiently small” in the sense required by moral theories that appeal to such risks (see Sect. 2). This renders quantified individual risks an implausible right- or wrong-making feature of the risk-imposing activities that we engage in while going about our daily lives. While some moral indeterminacy may well be a fact of life, it does not seem pervasive in the context of rather mundane activities such as walking a dog or driving a car.^31^

Against this, one might argue that we should understand the “conscientious and statistically well-versed reasoner” that plays an important role in my proposed interpretation of probability as someone who not merely consults and interprets existing data and studies, but who also goes about collecting relevant data and performing appropriate studies where such data and studies are missing. In this way, our ideal reasoner might generally be able to quantify the effects they deem potentially morally relevant.

I grant that we might get rid of much indeterminacy if we extend our understanding of the idealised reasoner in this way. At the same time, this understanding of the idealised reasoner seems overly demanding for the purposes of subjective moral principles. After all, such principles aim to identify the right- or wrong-making features of an action in light of the evidence available to an agent at the time of acting. In this way, it seems to be their main purpose to clarify what it means for an agent to take into account, in a morally appropriate manner, the information that they have access to at the time of acting. If we employ the understanding of the idealised reasoner that is needed to avoid indeterminacy, it follows that ordinary agents are asked to emulate a reasoner with capacities that are decidedly out of their reach. Getting rid of the worry of indeterminacy thus comes at the cost of an overly demanding interpretation of what it means for evidence to be “available” to us in our role as agents.

Let me conclude this section with a clarification. My critical discussion has relied heavily on the insight that there is usually a gap between the factors an idealised reasoner deems potentially relevant to some issue and the ones whose effects the reasoner is able to quantify based on existing data and studies. Importantly, this is *not* the reference class problem that plagues the frequency interpretation of probability and, with it, the objectively constrained subjective interpretations of probability (including mine) that are committed to the idea that conscientious reasoners will rely on suitable frequentist data (see footnote 22). As stated in Sect. 3.1, according to frequentism, the probability that some event will occur is the relative frequency with which the type of event occurs in some relevant reference class. For the Pit Bull example, the probability that your walk will result in an accident is simply the percentage with which comparable walks result in an accident. The reference class problem is the problem of settling what should count as “comparable”.^32^ Should the reference class for your dog walking include all the people who (world-wide? nationwide?) walk their “dangerous breed” dogs? Or should it include only experienced dog owners who walk their Pit Bulls? Or should the reference class be formulated even more narrowly? Settling this issue in a compelling and systematic manner is crucial for frequentism, but no generally accepted solution has been proposed to date.^33^ In this paper, I assume that the right reference class includes *all the factors that an idealised reasoner deems potentially relevant in light of their general understanding of the phenomenon at issue*. The key problem that I draw attention to is *not* that an idealised reasoner will struggle to decide what these factors are—this, in essence, would be the reference class problem—but that the factors that the reasoner identifies will usually exceed the ones whose impact they are able to quantify. Of course, this does not mean that the reference class problem does not show up at all on my account of probability. As I see it, it is quite likely that an expert may sometimes be unsure whether some particular factor should be taken into account. Having said that, where there is such doubt, this could in principle be resolved if the expert had access to data that allowed them to estimate the causal contribution of the factor they are doubtful about. If a statistically significant contribution can be found, the factor should be included; otherwise, it should be discarded. In this sense, what gives rise to indeterminacy on my suggested interpretation of probability is the practical inability to quantify the impact of factors that are deemed potentially relevant, not an expert’s doubts about what these factors are.

## Doing away with quantified individual risks

If the issues that I have identified in Sect. 3 are to the point, then we should make no appeal to quantified individual risks when conceptualising morally permissible risk imposition. In this section, I briefly sketch what might replace such an appeal. I propose that principles of morally permissible risk imposition do well if they recognise both the *value* of attempting to quantify an activity’s riskiness, as well as the *practical limits* we face when attempting such quantifications. The principles should thus not do away completely with a quantitative element. At the same time, they should make allowance for the fact that by including a quantitative element, they include a consideration that we know will not in general be tailored to the specific circumstances of any particular agent. How principles of morally permissible risk imposition best achieve this balancing act will depend on the circumstances. In what follows, I briefly sketch the outlines of what I consider a promising strategy for cases where a risky activity is legally and conventionally regulated in a defensible manner. I hope that this will help clarify what an appropriate such balancing act might look like.^34^

Consider again the example of dog-walking. Over time, we have developed societal rules pertaining to this activity. Some such rules are embodied in the law; others are merely conventional. Many jurisdictions, for example, specify dog leashing requirements, or lay out procedures a dog owner is required to follow if their dog has shown aggressive behaviour. To be defensible, such societal *due care rules* or *duties of care* need to be based on quantitative estimates of the societal risk that dog owners pose through their pets. More precisely, society-level data needs to support the conclusions that (i) the societal risks associated with dog ownership are sufficiently low if dog owners generally abide by the stipulated duties of care and that (ii) there is no competing and readily implementable due care regime that would keep societal risks similarly low, while putting fewer constraints on the liberties of dog owners.

Where legal and conventional rules are based on quantitative estimates in this way, subjective moral principles can refer to them as generally binding not only in a legal or conventional sense, but also in a moral sense.^35^ From a moral point of view, however, deviations from the societal rules will sometimes be justified, as the societal rules are not designed to take into account the idiosyncrasies of particular agents. Subjective moral principles thus do well if they clarify that an agent’s idiosyncratic features can give rise to principled exceptions based on our general understanding of the phenomenon at issue. Consider the example of Stefanie, a K9 police officer who, upon retiring, adopts Bella, an elderly Labrador Retriever, from the local shelter. In some jurisdictions, Stefanie might be legally required to undergo basic training with Bella, her professional experience notwithstanding. If Stefanie chooses to skip this training because she lives far away from the closest training center, this might well be morally justifiable, as the training seems highly unlikely to provide Stefanie with tools or knowledge she will otherwise lack. To put the same point differently, a requirement to undergo basic training when acquiring a dog serves the purpose of building expertise in the dog owner. As Stefanie already has relevant expertise, she may morally permissibly disregard the relevant requirement as unnecessary, given her circumstances.

Going in the other direction, consider the example of Bill, an inexperienced prospective dog owner who considers acquiring Rocket, a Pit Bull who will otherwise be put down for having shown aggressive behaviour. Bill may well be able formally to abide by his society’s dog leashing requirements. The purpose of such requirements, however, is to ensure that dog owners are able to restrain their dogs. If Rocket is clearly capable of overpowering Bill—because Rocket might easily bolt, for example, if Bill fails to pay close attention—this speaks against acquiring Rocket. Bill would, after all, be able to abide only by the letter, but not by the spirit, of relevant societal rules.

Generally deferring to societal due care rules while making room for principled exceptions is a suitable strategy for subjective moral principles whatever their primary purpose. If they are meant primarily to guide action, principles that follow this strategy relieve agents of the sizeable burden of quantification by instructing them to take their cue from existing rules that are based on society-level quantifications. If, by contrast, it is the principles’ main aim to identify morally relevant considerations from an evidence-relative perspective, the principles clarify that an agent appropriately takes into account the usefulness of quantifying an activity’s riskiness if she generally defers to societal rules based on society-level quantifications, and considers departing from them only in cases where her particular circumstances support such a departure in light of the relevant due care rules’ intended purpose.

## Conclusion

In this paper, I have argued that we should not appeal to quantified individual risks in our accounts of morally permissible risk imposition. There is usually a gap between the factors that an agent plausibly deems relevant to the risks that she imposes on others by engaging in an activity in a particular manner, and the factors whose effects she is reasonably able to quantify. Whenever this gap is present, subjective moral principles that make reference to quantified individual risks fail to provide an agent with useful practical guidance. If the occurrence of such gaps is common, this moreover implies that according to subjective moral principles that make reference to quantified individual risks, it is frequently indeterminate whether engaging in a risky activity is morally permissible. This indeterminacy can be avoided only if we accept an implausibly demanding interpretation of what it means for a piece of evidence to be “available” to an agent at the time of acting. I have argued that, taken together, these considerations render quantified individual risks an unsuitable feature of subjective moral principles.

If the criticisms that I have put forward in this paper are valid, then we do well if we conceptualise morally permissible risk imposition in a way that does away with quantified individual risks. This does not mean, however, that quantitative approaches to regulating risk-imposing activities should be disregarded from a moral point of view. I have suggested that principles of morally permissible risk imposition do well if the acknowledge both the value of attempting to quantify an activity’s riskiness, as well as the practical limits we encounter when attempting such quantifications.

My focus throughout this paper has been on conceptualising morally permissible risk imposition in circumstances where an agent considers engaging in an activity that is generally deemed socially acceptable, and where the agent does not intend to inflict harm on anyone, but is saliently able to foresee that their actions might result in harm to uninvolved others. Having said that, if my discussion has been to the point, then it is clear that my main criticisms will generalise.^36^ More precisely, any decision that we take under realistic circumstances is essentially a decision under uncertainty, as we are not, in general, able to predict with certainty what will be the consequences of our actions. Moreover, it is almost always possible—though of course not always salient—that our actions might cause harm to others.

With respect to prudential decision-making, orthodox normative decision theory stipulates that we adequately take uncertainty about the consequences of our actions into account if we choose an action that maximises our *expected utility*. The expected utility of an action for an agent is the sum of the agent’s suitably quantified evaluations (“utilities”) of the action’s possible outcomes, *with each utility discounted by the probability with which the agent thinks the respective outcome will occur if she performs the action*.^37^ In normative ethics, maximising act consequentialists have readily taken the basic structure of orthodox normative decision theory on board, and have argued that an agent acts subjectively permissibly if, and only if, she chooses in a way that maximises the expected goodness of the consequences of her actions.^38^ If my key contentions in this paper are to the point, then maximising act consequentialists have to rethink their subjective theorising quite generally, as appeals to quantified individual risks are pervasive within the theorising as it is currently done.

Deontologists have not similarly assimilated the key claims of orthodox decision theory. Largely they have simply shunned discussions of risk and uncertainty, conducting their theorising in an idealised objective or “fact-relative”^39^ realm where it is assumed that an agent knows with certainty what will be the consequences of her actions.^40^ The underlying thought of this practice may be that even though it is idealising to assume that agents have perfect foresight, this idealising assumption is innocent (and, indeed, productive), roughly because we can always unproblematically combine objective moral principles with a theory for making decisions under uncertainty to obtain suitable subjective principles. An important emerging literature draws our attention to the fact that this is false. Given the structure and basic commitments of deontological theories, their objective principles cannot always be combined with a decision theoretic framework to yield appropriate subjective principles.^41^ Part of this literature even argues that it is conceptually impossible for deontological theories to deal with risk and uncertainty in an adequate manner, and that we should therefore reject such theories.^42^

If I am right, then maximising act consequentialists are in a significantly more uncomfortable position with respect to their subjective theorising than they tend to believe, and deontologists find themselves at much less of a comparative disadvantage. Instead, both types of theorists have a pressing reason to concern themselves with the challenging task of working out subjective principles that are genuinely sound. My own hunch is that from both a consequentialist and a deontological perspective, there is no basic structure for subjective principles that will fit all contexts, not least because some areas of our lives are quantitatively much better understood than others (cf. footnote 34). Be that as it may, it is evident that much work on the topic remains to be done.

Judith Thomson has once remarked that when it comes to moral issues connected to risk and uncertainty, “[t]he noise of the real is deafening”^43^ once we attempt to rid ourselves of idealising assumptions. This is surely right. We do no more than bury our heads in the sand, however, if we take this as a license to make no serious attempt at engaging with the real.

## Footnotes

1 See e.g., Gibbard (2005). Derek Parfit’s term for what I call *subjective* permissibility is permissibility in the *evidence-relative sense*. See Parfit (2011, p. 151).

2 Gibbard (2005). Parfit uses the term *fact-relative* instead of *objective*. See Parfit (2011, p. 150).

3 Smith (2018).

4 Väyrynen (2006) and Smith (2018). It is an important question how we can establish that some strategy will reliably help promote the best possible consequences if, as agents, we are not generally able to deliberatively work out which of our alternatives would be morally optimific. If the morally optimific option frequently becomes clear with the benefit of hindsight, or in light of other additional information that we are eventually able to acquire, then it may be possible to formulate reliable action-guiding strategies. But if our ignorance of what would promote the best possible consequences runs deeper, it may not be possible to test potential strategies for their reliability, in which case maximising act utilitarianism fails to be action-guiding even in an indirect manner. For an argument to the effect that our ignorance about the consequences of our actions runs hopelessly deep, see Lenman (2000).

5 See e.g., Jackson (1991).

6 See e.g., Thomson (1990).

7 See e.g., Scanlon (2008).

8 Ibid., pp. 47–52. See also Tenenbaum (2017).

9 For an important exception, see Tenenbaum (2017).

10 Nozick (1974, p. 73).

11 See e.g., James (2012), Kumar (2015) and Lenman (2008).

12 Lenman (2008, p. 114).

13 Lazar (2017, p. 580).

14 Ibid., p. 584.

15 See e.g., Gillies (2000).

16 Hájek (1997).

17 Coherent credences are credences that satisfy the probability calculus.

18 The judgements need to be responsive to evidence only in the thin sense that in situations where reasoners receive new evidence, subjective Bayesianism requires that they update their credences following a rule called *Bayesian updating*.

19 Hájek (2007, p. 577), original emphasis.

20 This is not to say that unreasonable beliefs are morally irrelevant *simpliciter*. If I try to kill you with an unloaded gun that I believe to be loaded, I am blameworthy for trying to kill you even if I could easily have verified the gun’s non-threatening status. Parfit talks about permissibility in the “belief-relative sense” to capture the idea that there is a morally relevant subjective perspective which is unconstrained by the available evidence (see Parfit, 2011, p. 150). This paper, however, sets aside such issues, and asks how we should conceptualise the permissibility of engaging in risky activities in light of the evidence available to an agent at the time of acting.

21 As I have presented it here, my preferred interpretation of probability shares important similarities with objective Bayesianism. While objective Bayesianism is not usually understood as an interpretation of probability, but as a formal epistemology that makes claims about what beliefs it is rational for us to adopt, it moves beyond subjective Bayesianism partly by demanding that we calibrate our credences to the available evidence. Crucially, however, objective Bayesians assume that the partial beliefs of an ideally rational reasoner are coherent credences, whereas it is one of my key arguments in this paper that a conscientious and statistically well-versed reasoner would frequently refuse to quantify her partial beliefs. For a seminal work on objective Bayesianism, see Williamson (2010).

22 That the key ideas of the frequency interpretation enter objectively constrained subjective interpretations of probability in this way is a common assumption defended e.g., in Colyvan et al. (2001, p. 175), Hájek (2007) and Oberdiek (2017).

23 Once you have decided what factors potentially affect the riskiness of your dog walking, there are two general ways in which you might estimate the agent-specific risk that you impose on others by walking Faustus. First, you can try to put together a statistically representative sample of dog walks that share your specific risk characteristics. If you are able to put together such a sample, the relative frequency with which accidents occur in this sample provides an unbiased estimate of the risk that you impose through your dog-walking. If you cannot construct such a sample (as will usually be the case if you rely on existing data), you might alternatively model the riskiness of a risk-imposing activity based on the factors that you deem relevant to the phenomenon, and—using data from a different reference class than the one sharing your specific risk characteristics—proceed to estimate the causal effects of the factors you have identified. This second strategy is less demanding with respect to data requirements, but more demanding with respect to statistical methods. If done correctly, it will also yield an unbiased estimate of the riskiness of your dog-walking.

24 Kraese satirises a very similar impression in her “Why Your Dog Needs to be Leashed During Walks (But Mine Does Not)” (2020). See also footnote 30.

25 Note that I am not saying here that relying on the notion of an average risk as it applies to some population is never admissible or useful. To give a simple example, grasping that a mountain lion is on average 95% likely to attack if you approach its young is a very useful piece of information that you are well-advised to take into account if you spot some mountain lion cubs in the wild. This holds true even if there are (and even if you know there are) two types of mountain lion, the very gentle ones who are extremely unlikely to attack, and the much more numerous confrontational ones who are virtually guaranteed to attack, such that it is not true of any particular mountain lion that its probability of attacking you is 0.95. What matters for your survival is not that you get a particular mountain lion’s idiosyncratic risk of attack right, but only that you understand what danger these animals pose on average. There is, in fact, a large literature that both proceeds from and aims to defend the assumption that human and at least some non-human animals are very apt at “intuitive statistics”, explicitly or implicitly using statistical syllogisms to assess situations and make predictions. For an important work that introduces these ideas in a pertinent manner, see Gigerenzer and Murray (2011 [1987]). I thank an anonymous reviewer for pushing me to clarify these issues.

26 I think this holds true both for decision-making where we aim not to impose excessive risks on others, as well as for situations where we are choosing primarily with an eye to financial profit, or to what it is otherwise prudentially optimal to do. That is, we generally—and reasonably—settle for data about a population where we have reason to assume that there is heterogeneity in risk profiles, and content ourselves with estimating the risk posed by an “average member” of such a heterogeneous population. Insurance providers, for example, reasonably rely on such average risk estimates because doing so is sufficient to ensure profitability if enough policies are sold, and if competitors do not make more fine-grained distinctions. Where our idiosyncratic risk profile is more pertinent (as happens to be the case, for example, if we are trying to establish whether we should commute to work by bicycle or by bus), we tend to simply accept that the data that we have access to is not tailored to our situation, and, accordingly, tend to regard it as one type of input among a variety of considerations that are not easily made commensurable.

27 Serious quantitative estimates of riskiness will include not only a “best guess” or point estimate, but will also specify an interval or range within which the true variable of interest is likely to fall. Depending on the circumstances, a conscientious and statistically well-versed reasoner will decide to form only imprecise credences based on the intervals identified by relevant studies, e.g, in an effort adequately to represent the uncertainty inherent in the problem. See also footnote 30.

28 Mellor (1971, p. 7), my emphasis.

29 I thank two anonymous reviewers for drawing my attention to this possibility.

30 It is, of course, quite tempting to assume that in cases where non-quantifiable characteristics pull in opposing directions, access to some relevant population average enables us to come up with at least an imprecise probability for our idiosyncratic risk. After all, the only thing that is needed in such a context is a suitable *adjustment* of the population average in light of our idiosyncratic risk characteristics. It might then seem that based either on a “winging it” strategy or, as an anonymous reviewer points out, our intuitive statistics capacity, we are in a position to make such an adjustment. Consider the “winging it” strategy first. In the dog walking example, it might seem quite “commonsensical” to conclude that if you keep your good-natured Pit Bull on a leash, the risk that you impose on others is “likely” to lie somewhere between 1 in 100,000 and 1 in 10 million. After all, there are factors that plausibly raise your idiosyncratic risk over the societal average (you own a Pit Bull), but also factors that plausibly have a mitigating effect (you always keep good-natured Faustus on a leash). It is therefore not completely ill-founded to assume that the risk that you impose neither exceeds nor falls below your societal average by more than one order of magnitude. Proceeding in this rough-and-ready way, however, has all (in Sect. 3.2) “winging it” strategies suffer from. Conjuring up imprecise estimates in the absence of a data-based investigation that supports them (see footnote 27) is thus not a strategy that a conscientious and statistically well-versed reasoner would resort to. Next, consider the idea that we are capable of making the necessary adjustments based on our intuitive statistics capacity, which allows us to capture a variety of phenomena in frequentist terms even in the absence of any articulable knowledge of statistics or its methods (cf. footnote 25). While the literature on the topic suggests that we are frequently surprisingly good intuitive statisticians, we have reason to doubt that this is true in contexts where we have to assess how we deviate from some population average. There is an extensive psychological and behavioural economics literature that documents the biases that shape our perceptions and decision-making. Most importantly for our purposes, the so-called “Dunning-Kruger effect” is best explained by the fact that we are prone to overestimate our competence precisely in areas where we lack expertise. The effect is robust, and has been replicated in hundreds of studies (see e.g., Ehrlinger et al. 2008). There is also the widely studied phenomenon of “overconfidence” or “optimism bias”, according to which we systematically overestimate the likelihood with which our endeavours will succeed (see e.g., Sharot 2011). We thus have good reason to doubt that we are, in general, competent judges of the extent to which (or even the direction in which) we tend to differ from the rest.

31 According to John Oberdiek, we should interpret the probability of some harm occurring as a result of some particular action as the *maximum credence a reasonable agent would assign to the harm’s occurrence if the relevant action is performed*. Oberdiek thus puts forward an objectively constrained subjective interpretation of probability for subjective moral principles that differs from the one I put forward in this paper (see Sect. 3.1). Unlike the interpretation that I propose, Oberdiek’s moralises risk by building caution into the very definition of probability. As I read his account, reference to a *maximum reasonable credence* is introduced not only to moralise the notion of the riskiness of an action, but also in an attempt to ensure that the probability of a harmful event will usually be sufficiently determinate. See Oberdiek (2017), especially chs. 1 and 2. I believe, however, that we cannot successfully escape the issues that I raise in this paper by accepting Oberdiek’s account of probability. By referring to the idea of a reasonable maximum credence, Oberdiek simply presupposes that it will always be reasonable to adopt quantitative beliefs about agent-specific risks. It is my key contention, however, that this presupposition is false.

32 Gillies (2000, esp. 97) and Hájek (2007).

33 A prominent proposed solution is due to John Venn and Hans Reichenbach, who argue that the relevant reference class is “the narrowest class for which reliable statistics can be compiled” (Reichenbach, quoted in Hájek (2007, p. 568). I find this solution lacking because it simply disregards the gap between factors that we plausibly deem relevant in light of our causal understanding of some phenomenon, and the ones for which it happens to be the case that “reliable statistics can be compiled.” Adler (2003) discusses risk imposition from a legal perspective at pp. 1344–1349, and concludes that Reichenbach and Venn’s solution to the reference class problem is satisfactory for legal purposes. John (2014, pp. 36–43) and Kumar (2015) defend moralised solutions to the reference class problem. Fried (2020, argues pp. 111–120) that the reference class problem introduces indeterminacy to risky situations that contractualists in particular have hitherto failed to pay attention to.

34 It is not always true that risky activities are legally and conventionally regulated in a defensible manner. Consider, for example, the ill-understood and initially unregulated risks of a Covid-19 infection that we imposed on each other by going about our daily lives towards the beginning of the pandemic. It is not my aim, in this paper, to settle how we should think about the moral permissibility of engaging in such ill-understood activities. It seems clear, however, that appealing to quantified individual risks will not do, essentially for the reasons laid out in Sects. 2 and 3 of this paper. I thank an anonymous reviewer for highlighting this issue.

35 For a general argument in favour of the claim that we should sometimes regard legal and conventional rules as morally binding, see Honoré (1993).

36 I thank an anonomyous reviewer for drawing my attention to this point, and for pushing me to acknowledge it in the context of this paper.

37 See e.g., Pettigrew (2020, esp. 14-1-5).

38 See e.g., Jackson (1991).

39 Parfit (2011, p. 150).

40 For important exceptions, see Lazar (2017).

41 See Colyvan et al. (2010), and also Brown (2011).

42 See Fried (2020) and Jackson and Smith (2006).

43 Thomson (1986).
